# Current clinical practice in the management of phyllodes tumors of the breast: an international cross-sectional study among surgeons and oncologists

**DOI:** 10.1007/s10549-023-06896-1

**Published:** 2023-03-06

**Authors:** Carl Sars, Helena Sackey, Jan Frisell, Paul W. Dickman, Fredrik Karlsson, Isabelle Kindts, Gustavo Nader Marta, Ruffo Freitas-Junior, Tove Filtenborg Tvedskov, Loay Kassem, Ahmed S. Ali, Hanna Ihalainen, Mathias Neron, Michalis Kontos, Orit Kaidar-Person, Icro Meattini, Anne Brecht Francken, Frederieke van Duijnhoven, Ingvild Ona Moberg, Tanja Marinko, Attila Kollar, Mahbubl Ahmed, Dennis Remoundos, Jenny Banks, Reshma Jagsi, Lesly A. Dossett, Ebba K. Lindqvist

**Affiliations:** 1 Department of Molecular Medicine and Surgery, Karolinska Institutet, 171 77 Stockholm, Sweden; 2 Division of Cancer, Department of Breast, Endocrine Tumors and Sarcoma, Karolinska University Hospital Stockholm, Stockholm, Sweden; 3 Department of Medical Epidemiology and Biostatistics, Karolinska Institutet, Stockholm, Sweden; 4 General Hospital Groeninge, Kortrijk, Belgium; 5 Department of Radiation Oncology, Hospital Sírio-Libanês, São Paulo, Brazil; 6 CORA Advanced Center for Diagnosis of Breast Diseases, Hospital das Clínicas, Federal University of Goias, Goiânia, Brazil; 7 Department of Breast Surgery, Gentofte Hospital, Copenhagen, Denmark; 8 Department of Clinical Oncology, Cairo University Hospitals, Cairo, Egypt; 9 Comprehensive Cancer Center, University of Helsinki and Helsinki University Hospital, Helsinki, Finland; 10 Institut du Cancer de Montpellier, Surgical Oncology Department, Université Montpellier, Montpellier, France; 11 1st Department of Surgery, Laiko Hospital, National and Kapodistrian University of Athens, Athens, Greece; 12 Breast Radiation Unit, Sheba Tel Hashomer, Ramat Gan, Israel; 13 Sackler School of Medicine, Tel-Aviv University, Tel-Aviv, Israel; 14 Department of Experimental and Clinical Biomedical Sciences “M. Serio”, University of Florence, Florence, Italy; 15 Radiation Oncology Unit, Oncology Department, Azienda Ospedaliero Universitaria Careggi, Florence, Italy; 16 Department of Surgery, Isala Clinics, Zwolle, The Netherlands; 17 Netherlands Cancer Institute, Antoni Van Leeuwenhoek Hospital, Amsterdam, The Netherlands; 18 Department of Breast and Endocrine Surgery, Oslo University Hospital Ullevål, Oslo, Norway; 19 Institute of Oncology, Ljubljana, Slovenia; 20 Medical Faculty, University of Ljubljana, Ljubljana, Slovenia; 21 Department of Medical Oncology, Inselspital, Bern University Hospital, Bern, Switzerland; 22 University College London Hospitals NHS Foundation Trust, London, UK; 23 Oxford University Hospitals NHS Foundation Trust, Oxford, UK; 24 South West Peninsula Deanery, Devon, UK; 25 Department of Radiation Oncology, University of Michigan, Ann Arbor, USA; 26 Department of Surgery, University of Michigan, Ann Arbor, USA; 27 Department of Clinical Science and Education, Stockholm South General Hospital, Karolinska Institutet, Stockholm, Sweden; 28 Department of Surgery, Stockholm South General Hospital, Stockholm, Sweden

**Keywords:** Phyllodes tumor, Breast neoplasms, Margins of excision, Surgical oncology

## Abstract

**Purpose:**

Phyllodes tumors of the breast are rare fibroepithelial lesions that are classified as benign, borderline or malignant. There is little consensus on best practice for the work-up, management, and follow-up of patients with phyllodes tumors of the breast, and evidence-based guidelines are lacking.

**Methods:**

We conducted a cross-sectional survey of surgeons and oncologists with the aim to describe current clinical practice in the management of phyllodes tumors. The survey was constructed in REDCap and distributed between July 2021 and February 2022 through international collaborators in sixteen countries across four continents.

**Results:**

A total of 419 responses were collected and analyzed. The majority of respondents were experienced and worked in a university hospital. Most agreed to recommend a tumor-free excision margin for benign tumors, increasing margins for borderline and malignant tumors. The multidisciplinary team meeting plays a major role in the treatment plan and follow-up. The vast majority did not consider axillary surgery. There were mixed opinions on adjuvant treatment, with a trend towards more liberal regiments in patients with locally advanced tumors. Most respondents preferred a five-year follow-up period for all phyllodes tumor types.

**Conclusions:**

This study shows considerable variation in clinical practice managing phyllodes tumors. This suggests the potential for overtreatment of many patients and the need for education and further research targeting appropriate surgical margins, follow-up time and a multidisciplinary approach. There is a need to develop guidelines that recognize the heterogeneity of phyllodes tumors.

## Introduction

Phyllodes tumors of the breast are rare fibroepithelial lesions that are classified according to their morphology as benign, borderline or malignant [1, 2]. They entail a broad range of pathological and clinical features and therefore regarded as a spectrum of fibroepithelial neoplasms rather than a single entity. For individuals with malignant phyllodes tumors the prognosis is poor when metastasis occur [3]. In contrast, long-term prognosis for borderline phyllodes tumors is more favorable, and mortality from benign tumors are close to zero. In addition, benign phyllodes tumors show a low recurrence rate and no progression of histology when tumours recur [4–6].

Early diagnosis is essential to enable timely surgery, as most phyllodes tumors are fast-growing [7]. The diagnostic process for phyllodes tumors is complicated by their similarity of benign phyllodes tumors to benign fibroadenomas and the similarity of borderline or malignant phyllodes tumors to soft-tissue sarcomas [8–12]. The diagnostic challenge comprises overlapping microscopic features and sampling limitations of core-needle biopsy (CNB) [13, 14]. Triple assessment (i.e. physical examination of the breasts, breast imaging and a biopsy/cytology), which is standard work-up for a lump in the breast, has been shown to have low diagnostic accuracy in phyllodes tumors [15]. The work-up is thus often protracted by the above-mentioned complicating factors, and a final diagnosis is sometimes not determined until after the pathology report from the resection.

Surgical resection, in the form of lumpectomy or mastectomy (without axillary staging), is the accepted mainstay of treatment [12, 16], but there is no international consensus on appropriate surgical margins or regarding indications for adjuvant therapy. As phyllodes tumors rarely metastasize to the lymph nodes, there is no benefit of axillary staging in these cases [17]. Overall, reported recurrence rates are 10–17% for benign, 14–25% for borderline, and 23–30% for malignant phyllodes tumors, and recurrence is more common after incomplete resection [18]. Indeed, in a recent multi-institutional study of 550 phyllodes tumors, 42% (*n* = 231) had a positive surgical margin and a second resection was performed in 51 patients with an initial negative, yet narrow, surgical margin (82.4% < 2 mm) [19]. The local recurrence rate at a median follow of 36.7 months was only 2.7% (*n* = 2) among those with positive surgical margins who did not undergo a repeat excision and residual tumor burden was low in those who did, suggesting that wide margins might not always be required [19]. The authors concluded that evidence-based guidelines for the surgical management of phyllodes are needed [19].

The role and requirements of specialized and comprehensive breast cancer centers is well described and involves a multidisciplinary approach in the clinical setting, as well as research opportunities [20]. Nevertheless, there is no international consensus on whether phyllodes tumors should be managed at breast cancer centers or via sarcoma units, nor on imaging modality, surgical margins, adjuvant treatment, follow-up length and intervals. Various strategies are used for diagnosis of patients with phyllodes tumors, including fine-needle aspiration (FNA), CNB, radiology and clinical examination [15, 21, 22]. Moreover, only a few countries have national guidelines for treatment of phyllodes tumors [12, 16, 23]. The North American National Comprehensive Cancer Network (NCCN) and the MD Anderson Cancer Center in the US have guidelines on breast tumors that includes recommendations on the diagnosis and treatment of phyllodes tumors, although they do not build upon randomized controlled studies and their use in international clinical practice is unknown [12, 16]. These guidelines, as well as guidelines from Collège National des Gynécologues et Obstétriciens Francais (CNGOF) in France, are summarized in Table 1.

**Table 1:** Summary of national guidelines on the management of phyllodes tumors from the US and France

	National guidelines on the management of phyllodes tumors		
	MD Anderson [12]	NCCN [16]	CNGOF [23]
FNA	No (Often inconclusive)	No (Often inconclusive)	Not stated
CNB	Yes	Yes	Yes
Surgery	Wide excision	Wide excision	Wide excision
Margins	Not stated	Excisional biopsy in benign, 10 mm margin in borderline and malignant	Clear in benign, 10 mm in borderline
Axillary staging	No	No	Not stated
Adjuvant RT	Consider if malignant	In certain settings	Not stated
Adjuvant Chemotherapy	Not stated	Not stated	Not stated
Follow-up	Observe, length not specified	3 years	Not stated

*FNA* Fine Needle Aspiration, *CNB* Core needle biopsy, *RT* radiation therapy, *NCCN* National Comprehensive Cancer Network, *CNGOF* Collège National des Gynécologues et Obstétriciens Francais

In light of the limited consensus regarding the management of phyllodes tumors, and lack of evidence-based guidelines, we conducted a survey in order to describe current clinical practice in the diagnostic work-up, treatment, and follow-up of phyllodes tumors.

## Methods

The study was discussed during multidisciplinary meetings of the locoregional breast group, which is part of the European Organization for Research and Treatment of Cancer (EORTC) Breast Cancer Group. We conducted an international survey targeting surgeons working within the field of breast cancer and/or sarcoma surgery, and oncologists working within the field of radiation therapy and/or breast oncology. We used a cross-sectional approach allowing comparisons across medical specialties, individual experience, hospital types, countries, and continents. Through the EORTC Breast Cancer Group as well as through the professional networks of the authors, invitations were sent out to participate as investigators in the study. One to two national collaborators were appointed; one oncologist and/or one surgeon. The national collaborators were responsible for the distribution of the survey in their country as well as for the ethical review process, as specified below.

In terms of study design and reporting, we followed the Checklist for Reporting Results of Internet E-Surveys (CHERRIES).

### Survey instrument

A pilot survey was created by the coordinating investigating team (EKL, HS, CS, and JF) in order to address different decision-making steps in the work-up, management, and follow-up of individuals with suspected or confirmed phyllodes tumors of the breast. The survey was tested among twelve Swedish surgeons and oncologists for comprehension and clinical relevance, and was revised after completion. The collaborators from each participating country were then invited to further review the survey to ensure international applicability.

The final version of the survey was created, collected and managed in REDCap (Research Electronic Data Capture tool) [24, 25] hosted at Karolinska Institutet. REDCap is a secure, web-based software platform designed to support data capture for research studies. The survey language was English.

The national collaborators were asked to distribute the survey to surgeons working within the field of breast and/or sarcoma surgery, and/or to oncologists working within the field of radiation therapy and/or breast oncology. Lists of eligible participants were constructed before the dissemination of the survey, and the number of eligible participants/respondents of the survey invitation submitted to the coordinating investigating team.

In each participating country, the survey was distributed sometime between July and December 2021 by email. The respondents were informed they had one month to complete the survey, and during this time they received two reminders via email.

The data management and information provided was in accordance with the European Union’s General Data Protection Regulation (GDPR) [26]. No incentives were offered in exchange of completion of the survey.

The survey consisted of 135 questions in total, covering four domains; demographic information about respondents (country of employment, medical specialty, level of specialization, type of institution, volume of phyllodes patients, and referral routines), investigation and diagnostics, treatment, and follow-up. All questions were single-select or multiple-select multiple-choice. All items were non-mandatory, and all had a non-response option. Responses to multiple-choice questions with multiple-select answers were excluded if the respondent had selected the non-response option checkbox, in addition to other answer checkboxes. Some questions were only given as follow-up depending on a particular answer to a previous question. As a final query we asked participants an open-text question about their perceived knowledge gaps or lack of clinical evidence. A complete version of the survey is available in the Supplementary material (see Appendix1). The survey opened on 1 July 2021 and closed on 28 February 2022.

### Statistical analysis

Data were extracted from REDCap, and all data management and statistical analysis was performed in StataIC 13 (StataCorp, College Station, TX). Data was presented through descriptive analyses as frequencies, percentages and distributions. We performed pre-specified sub-analyses on all single-select multiple-choice items to see whether responses differed between more experienced and less experienced clinicians and/or by hospital type. For sub-analyses by clinician expertise, responses were divided into two categories based on number of cases of phyllodes that respondents had stated they had personally been involved in the management of in the last year (non-expert < 5 cases of benign, borderline, and/or malignant phyllodes tumors, versus expert ≥ 5 cases). For sub-analyses by hospital type, responses from respondents who stated they work at a university hospital were compared against responses from respondents who work at non-university hospitals (regional hospital or at an unspecified hospital type).

For comparisons between groups, Pearson’s chi square test was used. For all analyses, a *p*-value < 0.05 was considered statistically significant.

### Ethical approval

The study was approved by the Swedish Ethical Review Authority (Dnr 2020-03335), the Finnish Ethical Review Authority, the Brazilian Ethical Review Authority (CAAE:47661921.9.0000.5461/number 4.787.791), and the American Ethical Review Authority. For additional participating countries, ethical permit was waived according to national regulations and/or local ethical review boards. Written consent of respondents was collected through a compulsory box at the beginning of the survey.

## Results

A total of 546 responses were collected, whereof 127 were excluded due to the participant not providing their name and/or their signature as consent for participation and data analysis. All remaining 419 survey responses from 16 countries were analyzed after pseudonymization, regardless of missing information or incompleteness.

### Demographics

A total of 419 surgeons (60.1%) and oncologists (31.5%) participated in the survey, 35 participants (8.4%) did not specify medical specialty. The majority of respondents (54.9%) had worked more than ten years after specialization. Details of participants’ demographics are outlined in Table 1. From responding surgeons, 52.6% worked at a university hospital, 34.4% at a regional hospital and 12.6% at other institutions. The corresponding rates for oncologists were 62.7%, 25.4% and 11.9%, respectively.

### Investigations and diagnostics

When investigating a palpable breast lump 37.4% of respondents answered that a CNB always was indicated, 16.4% answered that a CNB was indicated when an FNA was inconclusive and 17.0% answered CNB was indicated when the FNA showed atypical cells. Another 21.9% of the participants claimed that they do not manage the work-up of this patient category.

In addition to CNB, ultrasound and mammography, 77.5% answered that investigation should be supplemented with magnetic resonance imaging (MRI) for the purpose of surgical planning. The corresponding rate for CT thorax was 19.8%.

Whereas 71.5% of participants state that adenocarcinoma of the breast is routinely discussed at a breast multidisciplinary team meeting (MDT), 44.5%, 63.0%, and 67.8% respectively state that benign, borderline, and malignant phyllodes tumors are routinely discussed. In regards to timing of the MDT, 26.7% of the respondents answer that the patients should be discussed after the initial biopsy (but before surgical resection) only, 6.2% answered after surgical resection only, 64.2% answered both before and after the surgical resection, 0.9% at another time point, and 2.1% did not have access to an MDT.

Type of breast surgery to be performed in malignant phyllodes tumors (e.g., mastectomy or breast-conserving surgery) is decided by the MDT in 71.5% of participants’ units and by the individual surgeon in 28.5% of units. The corresponding rates for borderline phyllodes tumors are 61.0% and 39.0%, respectively. For benign phyllodes tumors the corresponding rates are 50.4% and 49.6%, respectively.

### Surgical treatment

Regarding acceptable interval for excision, the most common answer for benign phyllodes tumors was within 3 months (28.2%), for borderline phyllodes tumors within 4 weeks (35.3%), and within 2 weeks (36.9%) for malignant phyllodes tumors.

Opinions on surgical resection margins are summarized in Table 2. The most common macroscopic intra-operative margins the surgeon should aim for in benign phyllodes tumors are “no tumor on ink” (36.3% of respondents), and for borderline and malignant phyllodes tumors 10 mm margin (39.6% and 42.5%, respectively).

**Table 2:** Demographics of survey participants

Variable	Total responses, *N* (%)
Respondents	419 (100)
Country of practicing	
Belgium	5 (1.2)
Brazil	118 (28.2)
Denmark	10 (2.4)
Egypt	13 (3.1)
Finland	24 (5.7)
France	36 (8.6)
Greece	2 (0.5)
Israel	10 (2.4)
Italy	11 (2.6)
Netherlands	24 (5.7)
Norway	27 (6.4)
Slovenia	13 (3.1)
Sweden	41 (9.8)
Switzerland	5 (1.2)
United Kingdom	18 (4.3)
USA	20 (4.8)
Surgical specialty	252 (60.1)
Breast surgery	160 (63.5)
Breast and Endocrine surgery	37 (14.7)
Sarcoma surgery	13 (5.2)
Sarcoma and Endocrine surgery	6 (2.4)
General surgery	2 (0.8)
Breast and General surgery	25 (9.9)
Plastic surgery	9 (3.6)
Oncologic specialty	132 (31.5)
Radiation oncology	92 (69.7)
Breast oncology	11 (8.3)
Sarcoma oncology	20 (15.2)
General oncology	7 (5.3)
Other	2 (1.5)
Data missing	35 (8.4)
Level of specialization	
Trainee/resident/fellowship	7 (1.7)
< 1 year as a specialist	5 (1.2)
1–3 years as a specialist	24 (5.7)
3–5 years as a specialist	40 (9.6)
5–10 years as a specialist	82 (19.6)
> 10 years as a specialist	230 (54.9)
Data missing	31 (7.4)
Number of breast cancer patients at institutional level (yearly)	
< 250	72 (17.2)
250–1000	222 (53.0)
> 1000	75 (17.9)
Data missing	50 (11.9)
Institution manages benign phyllodes	
Yes	348 (83.1)
No	33 (7.9)
Data missing	38 (9.1)
Institution manages borderline phyllodes tumors	
Yes	354 (84.5)
No	30 (7.2)
Data missing	35 (8.4)
Institution manages malignant phyllodes tumors	
Yes	339 (80.9)
No	41 (9.8)
Data missing	39 (9.3)
Personally involved in management of any phyllodes tumor in the last year	
Yes, < 5	262 (68.8)
Yes, ≥ 5	73 (19.2)
No	44 (11.6)
No answer/don't know	1 (0.3)
Missing data	1 (0.3)

In regards to microscopic margins, after final pathology report, 58.6% of respondents answered “no tumor on ink” was acceptable for benign tumors. However, for borderline tumors, only a minority (26.4%) considered “no tumor on ink” to be sufficient to avoid re-excision. For malignant phyllodes tumors the acceptance for a smaller surgical margin was even lower, with only 11.7% of respondents accepting “no tumor on ink” whereas 34.5% of respondents indicating that they would expect a 10 mm margin or more to avoid re-excision (Table 3).

**Table 3:** Opinions on surgical treatment of borderline and malignant phyllodes tumors

Variable	Total responses, *N* (%)	Variable	Total responses, N (%)
Respondents	419 (100)		
What macroscopic intra-operative margin should the surgeon aim for in cases of malignant phyllodes?	308 (73.7)^a^	What microscopic resection margin would you accept in order not to consider re-excision for a malignant phyllodes tumor?	307 (73.3)^b^
Free margins/no tumor on ink	11 (3.6)	Free margins/no tumor on ink	36 (11.7)
1 mm	7 (2.3)	1 mm	34 (11.1)
5 mm	26 (8.4)	5 mm	46 (15.0)
10 mm	131 (42.5)	10 mm	106 (34.5)
15 mm	9 (2.9)	15 mm	3 (1.0)
20 mm	61 (19.8)	20 mm	31 (10.1)
30 mm	11 (3.6)	30 mm	3 (1.0)
50 mm	11 (3.6)	50 mm	4 (1.3)
What macroscopic intra-operative margin should the surgeon aim for in cases of borderline phyllodes?	308 (73.7)^a^	What microscopic resection margin would you accept in order not to consider re-excision for a borderline phyllodes tumor?	307 (73.3)^b^
Free margins/no tumor on ink	33 (10.7)	Free margins/no tumor on ink	81 (26.4)
1 mm	24 (7.8)	1 mm	49 (16.0)
5 mm	50 (16.2)	5 mm	55 (17.9)
10 mm	122 (39.6)	10 mm	71 (23.1)
15 mm	8 (2.6)	15 mm	2 (0.7)
20 mm	32 (10.4)	20 mm	14 (4.6)
30 mm	4 (1.3)	30 mm	0 (0.0)
50 mm	2 (0.7)	50 mm	0 (0.0)

^a^Out of 419 participants, there were 308 responses and 111 who were non-responders or opted-out of responding

^b^Out of 419 participants, there were 307 responses and 112 who were non-responders or opted-out of responding

Regarding axillary surgery for benign phyllodes, 2.9% of respondents would recommend sentinel lymph node biopsy (SLNB) in case of no clinical or radiological suspected axillary metastases, 97.1% would refrain from any axillary surgery. The corresponding rate of recommending SLNB for borderline tumors, was 6.8%. In patients with in malignant phyllodes tumors, 18.6% of respondents would recommend a SLNB, 2.0% would recommend a routine axillary lymph node dissection (ALND) and 79.4% would not recommend any axillary surgery.

### Adjuvant treatment

For benign phyllodes tumors, 88.0% of the respondents would never recommend radiation therapy and for borderline and malignant phyllodes tumors the corresponding rates were 60.3% and 11.0% respectively. Overall, 98.3% of respondents would never recommend chemotherapy for patients with benign phyllodes tumors, the corresponding rates for patients with borderline and malignant phyllodes tumors were 96.6% and 57.5%, respectively. A total of 25.4% of respondents would recommend chemotherapy in the setting of malignant phyllodes tumors with elevated Ki-67 (MIB-1) proliferative index expression. Opinions on adjuvant radiation therapy and chemotherapy are summarized in Tables 4 and 5, respectively.

**Table 4:** Opinions on indications for adjuvant treatment

	When should patients with phyllodes tumors be recommended adjuvant chemotherapy?		
	Benign, *n* (%)^a^	Borderline, *n* (%)^b^	Malignant, *n* (%)^c^
Never	284 (98.3)	258 (96.6)	111 (57.5)
If breast conserving surgery is performed	3 (1.0)	3 (1.1)	7 (3.6)
If the resection margin is positive (tumor on ink) and further surgery is not possible	2 (0.7)	4 (1.5)	26 (13.5)
If the resection margin is unsatisfying and further surgery is not possible	2 (0.7)	2 (0.7)	17 (8.8)
If the primary tumor is/was 20–50 mm in greatest diameter	2 (0.7)	1 (0.4)	12 (6.2)
If the primary tumor is/was ≥ 50 mm in greatest diameter	3 (1.0)	5 (1.9)	50 (25.9)
If the tumor has elevated Ki-67 (MIB-1) expression	N/A	N/A	49 (25.4)

	When should patients with phyllodes tumors be recommended adjuvant radiation therapy?		
	Benign, *n* (%)^d^	Borderline, *n* (%)^e^	Malignant, *n* (%)^f^
Never	257 (88.0)	161 (60.3)	28 (11.0)
If breast conserving surgery is performed	9 (3.1)	27 (10.1)	121 (47.5)
If the resection margin is positive (tumor on ink) and further surgery is not possible	29 (9.9)	80 (30.0)	142 (55.7)
If the resection margin is unsatisfying and further surgery is not possible	17 (5.8)	63 (23.6)	146 (57.3)
If the primary tumor is/was ≥ 50 mm in greatest diameter	5 (1.7)	19 (7.1)	101 (39.6)

^a^289 total respondents, 113 non-respondents and 17 opted-out of responding

^b^267 total respondents, 113 non-respondents and 39 opted-out of responding

^c^193 total respondents, 113 non-respondents and 113 opted-out of responding

^d^292 total respondents, 112 non-respondents and 15 opted-out of responding

^e^267 total respondents, 112 non-respondents and 40 opted-out of responding

^f^255 total respondents, 112 non-respondents and 52 opted-out of responding

**Table 5:** Volume and dosing of adjuvant radiation therapy in borderline and malignant phyllodes according to medical and radiation oncologists

What volume of RT would you recommend for borderline phyllodes?	Respondents *n* (%) 94 (100)	What volume of RT would you recommend for malignant phyllodes?	Respondents *n* (%) 94 (100)
Whole breast	37 (39.4)	Whole breast	40 (42.6)
Partial breast (tumor bed)	4 (4.3)	Partial breast (tumor bed)	9 (9.6)
Site of close margins only	1 (1.1)	Site of close margins only	1 (1.1)
Whole breast + boost to tumor bed	14 (14.9)	Whole breast + boost to tumor bed	30 (31.9)
Would never recommend RT	17 (18.1)	Would never recommend RT	1 (1.1)
Don’t know	21 (22.3)	Don’t know	13 (13.9)
If delivered, what dose of RT would you recommend for borderline phyllodes?	56 (100)	If delivered, what dose of RT would you recommend for malignant phyllodes?	80 (100)
60 Gy in 30 fractions	12 (21.4)	60 Gy in 30 fractions	25 (31.3)
50 Gy in 24 fractions	21 (37.5)	50 Gy in 24 fractions	27 (33.8)
40 Gy in 15 fractions	21 (37.5)	40 Gy in 15 fractions	21 (26.3)
26 Gy in 5 fractions	1 (1.8)	26 Gy in 5 fractions	1 (1.3)
Don’t know	1 (1.8)	Don’t know	6 (7.5)

These survey-questions were only given to those who were working in the field of medical or radiation oncology

*RT* radiation therapy, *Gy* Gray

### Follow-up

Recommended follow-up duration for all types of phyllodes tumors is most commonly selected as 5 years (Table 6).

**Table 6:** Opinions on follow-up length and method after treatment for phyllodes tumors

Variable	Total responses, *N* (%)^a^	Variable	Total responses, *N* (%)^a^
How long would you recommend follow-up for a patient who has been treated for a benign phyllodes tumor?	212 (100)	What imaging would you recommend in benign tumors?	205 (100)
3 months	5 (2.4)	Mammography	127 (62.0)
6 months	28 (13.2)	Ultrasound	152 (74.1)
1 year	28 (13.2)	Chest X-ray	1 (0.5)
2 years	41 (19.3)	CT Thorax	2 (1.0)
3 years	17 (8.0)	Full body CT	0 (0.0)
5 years	68 (32.1)	Full body PET-CT	2 (1.0)
10 years	7 (3.3)	MRI Breast	15 (7.3)
> 10 years	7 (3.3)	Full body MRI	0 (0.0)
Don't know	11 (5.2)	Clinical examination only, unless suspected recurrence	28 (13.7)
		Don't know	4 (2.0)
How long would you recommend follow-up for a patient who has been treated for a borderline phyllodes tumor?	255 (100)	What imaging would you recommend in borderline tumors?	252 (100)
3 months	14 (5.5)	Mammography	160 (63.5)
6 months	22 (8.6)	Ultrasound	179 (71.0)
1 year	7 (2.8)	Chest X-ray	14 (5.6)
2 years	30 (11.8)	CT Thorax	13 (5.2)
3 years	19 (7.5)	Full body CT	4 (1.6)
5 years	130 (50.1)	Full body PET-CT	2 (0.8)
10 years	18 (7.1)	MRI Breast	31 (12.3)
> 10 years	10 (3.9)	Full body MRI	0 (0.0)
Don't know	5 (2.0)	Clinical examination only, unless suspected recurrence	31 (12.3)
		Don't know	5 (2.0)
How long would you recommend follow-up for a patient who has been treated for a malignant phyllodes tumor?		What imaging would you recommend in malignant tumors?	256 (100)
3 months	23 (8.9)	Mammography	162 (63.3)
6 months	8 (3.1)	Ultrasound	151 (59.0)
1 year	0 (0.0)	Chest X-ray	30 (11.7)
2 years	7 (2.7)	CT Thorax	84 (32.8)
3 years	4 (1.6)	Full body CT	25 (9.7)
5 years	124 (48.1)	Full body PET-CT	10 (3.9)
10 years	60 (23.3)	MRI breast	59 (23.0)
> 10 years	27 (10.5)	Full body MRI	0 (0.0)
Don't know	5 (2.0)	Clinical examination only, unless suspected recurrence	27 (10.5)
		Don't know	2 (0.8)

^a^Percentages are of those who responded to each survey-question from among 419 total respondents

### Sub-analysis by clinician expertise

The responses to the question of what macroscopic intra-operative margin to aim for in surgery differed for benign phyllodes tumors according to two categories of clinician expertise (*p* < 0.05). For benign phyllodes tumors, 51.6% of expert clinicians versus 38.9% of non-expert clinicians would aim for no tumor on ink or only a 1 mm margin.

Opinions on what microscopic resection margin to accept in order not to consider re-excision differed for borderline phyllodes tumors according to clinician expertise (*p* < 0.05). For borderline phyllodes tumors, 51.6% of expert clinicians versus 40.0% of non-expert clinicians would accept either no tumor on ink or only a 1 mm margin on the pathology report. For malignant phyllodes tumors, 62.9% of expert clinicians versus 48.6% of non-expert clinicians would include the deep fascia (*p* < 0.05).

Opinions on whether to perform axillary surgery differed for malignant phyllodes tumors according to clinician expertise (*p* < 0.05). For malignant phyllodes tumors, 82.3% of expert clinicians versus 64.1% of non-expert clinicians would not do any axillary surgery.

### Sub-analysis by hospital type

The responses to the question on treatment decision differed for all types of phyllodes by hospital type (*p* < 0.05). For malignant phyllodes tumors, decisions on treatment were made more often at MDT in university hospitals whereas in non-university hospitals, the decision is more often made by the treating surgeon.

Opinions on acceptable time intervals for excision differed according to hospital type for benign phyllodes tumors (*p* < 0.05). A total of 0.6% of respondents at university hospitals and 9.9% of respondents at non-university hospitals replied that excision should take place within two weeks.

The responses to the question of whether to include the deep fascia in the excision differed for benign phyllodes tumors according to hospital type (*p* < 0.05). For benign phyllodes tumors, 4.9% of respondents at university hospitals versus 14.1% of respondents at non-university hospitals would include the deep fascia.

## Discussion

In this international survey of 419 clinicians, we found that current clinical management of phyllodes tumors varies considerably. Furthermore, practices concerning surgical treatment differ according to both clinical expertise and hospital type.

As expected, access to a joint breast and sarcoma MDT is greater at university hospitals and notably 2% of respondents state that they do not have access to an MDT at all. The MDT plays a greater role in decision making when it comes to malignant phyllodes tumors compared to benign or borderline phyllodes tumors. Overall, however, phyllodes tumors are discussed at MDT less frequent than breast adenocarcinoma, although more often in university hospitals. Considering the low diagnostic accuracy in phyllodes tumors [15] as well as the paucity of clinical guidelines, one might have expected clinicians to defer to the MDT more often.

We found that, for phyllodes tumors with more aggressive tumor biology, respondents in general recommend a quicker surgical excision and a wider excision margin. The NCCN guidelines recommend a 10 mm margin across all phyllodes tumor types, but for malignant phyllodes tumors, 14.3% of clinicians in our survey would recommend aiming for a margin less than 10 mm, and 37.8% would accept a microscopic margin less than 10 mm on pathology report. The French CNGOF guidelines differentiates between borderline phyllodes tumors, where a 10 mm margin is advised, and benign phyllodes tumors, where clear (no tumor on ink) margins are deemed sufficient [23]. In fact, a recent multi-institutional study from the US showed that in patients with phyllodes tumors and positive margins where re-excision was not performed, very few (*n* = 2; 2.7%) suffered a local recurrence [19]. We found that more expert than non-expert clinicians tend to aim for smaller margins in benign phyllodes tumors, which might reflect a clinical experience of a low recurrence-rate in these tumors regardless of margins. Furthermore, for benign phyllodes tumors, we found that more respondents based in regional hospitals recommend a rapid surgical treatment as well as excision of the deep fascia. Taken together, these findings could suggest that clinicians with shorter experience or those working in regional hospitals take a more aggressive treatment approach to benign phyllodes tumors in particular. This in turn could be due to lack of or conflicting guidelines, and/or limited access to or use of MDT.

Axillary surgery for phyllodes tumors is considered by 20% of respondents, where expert clinicians had a more restrictive approach. None of the available guidelines recommend axillary staging [12, 16, 23]. One reason for this divergence could be the low diagnostic accuracy of work-up for phyllodes tumors, resulting in uncertainty regarding axillary involvement and may perform axillary surgery as a safety measure [17]. Other reasons could be a poor knowledge of guidelines, inexperience with these rare tumor types, and/or limited access to or use of MDT.

As expected, clinicians are more likely to recommend adjuvant treatment to patients with borderline or malignant phyllodes tumors. None of the available guidelines suggest chemotherapy for phyllodes tumors; however, radiation therapy might be recommended in certain settings according to the guidelines by NCCN and MD Anderson [, , 12, 16, 23]. Our survey shows that among medical and radiation oncologists, the likelihood of never endorsing radiation therapy was lower than when the broader sample was asked. With regards to the adjuvant treatment of phyllodes tumors, there is a paucity of strong evidence, which likely explains the divergent responses by the clinicians. A heterogenous administration of radiation therapy have been shown using data from the Surveillance, Epidemiology, and End Results Program (SEER) [17, 27]. In two systematic reviews and meta-analyses, results on adjuvant therapy in phyllodes tumors are conflicting and no randomized studies have been undertaken [28, 29].

We found that respondents suggest follow-up for phyllodes tumors for five years with, most frequently, mammography or ultrasound. This is in contrast to the NCCN guidelines which recommend three years of clinical follow-up, without specifying preferred imaging method [16]. We believe this discrepancy is due to existing follow-up protocols for adenocarcinoma of the breast, but also protocols for follow-up of other sarcomas (retroperitoneal/abdominal) being used in the absence of local clinical guidelines for phyllodes tumors.

A recent study of margin management and adjuvant therapy in phyllodes tumors by Diego et al. found that among American breast surgeons, the practice variation was high. They also report uncertainty regarding treatment decision making, and the authors highlight the need to develop more specific national guidelines, which is in concert with our findings [30]**.**

Results from our study show that many clinicians suggest MRI of the breasts to be part of the work-up (77.5%) and surveillance (12.3–23.0%). There are published articles that have studied the value of MRI in phyllodes diagnostics, but to our knowledge there are no studies published on the use of MRI in surveillance after treatment for phyllodes tumors [31, 32]. The high rate of preferred pre-operative MRI in the purpose of surgical planning may reflect local traditions for any breast conserving surgery.

One limitation in our study is the multiple-choice design with predetermined options, where other strategies for work-up, treatment or follow-up may have been missed. Due to the nature of these rare tumors, it is likely that only a handful of clinicians at each medical unit manage the care of these patients. Although national and regional specialists were invited to participate in this survey, it was widely distributed and the collected answers are likely to represent a heterogeneous group of clinicians with varied experience of, and interest in, phyllodes tumors of the breast. Considering that a large portion (28.2%) of responders were from Brazil, the results may reflect treatment protocols and traditions from certain countries. The remaining responding countries, however, were not considerably overrepresented (ranging 1.2–9.8%).

The strengths of our study include the international setting, the inclusion of several medical specialties, and the careful design of the questionnaire after testing for comprehension in a pilot study. To the best of our knowledge, this is the first study to describe the clinical practice in the diagnosis, treatment, and follow-up of patients with phyllodes tumors, and to relate clinical practice to current guidelines.

We hypothesized that the lack of evidence-based guidelines results in broadly different patterns of care for these patients globally. The findings of our study appear to confirm this. Due to the rare nature of phyllodes tumors, large, prospective studies are lacking. Randomized trials, needed to answer questions regarding appropriate surgical margins and adjuvant treatment, may be difficult in rare tumors, although research within other areas international collaborations have enabled successful research [33]. Additional large, well-designed, observational cohort studies can likely contribute to the evidence in these rare tumors, to further examine the association between surgical margins and recurrence, and the role of adjuvant treatment in long-term outcomes. In the meantime, we suggest the findings in our survey serve to inform empirical consensus guidelines.

In conclusion, according to clinicians within the fields of breast surgery and/or oncology internationally, there is considerable variability in the management of phyllodes tumors of the breast. This is likely due to the absence of international guidelines, as well as poor dissemination and/or low acceptance of national guidelines, which are very diverse and not precise for these types of tumors. While some of the current clinical management may be attributed to concern about more advanced disease in the absence of strong data, there may be an educational gap regarding current guidelines and appropriate practice. However it is notably difficult to obtain a correct pre-operative diagnosis from biopsy material [34], not least regarding stage (benign/borderline/malignant) and that fact might influence clinicians to advocate wider excisions in order to err on the side of caution. We suggest empirical consensus guidelines to be crafted, inspired by our findings and grounded in the currently available evidence.

## Data Availability

The datasets generated during and/or analysed during the current study are not publicly available due to the European Union General Data Protection Regulation.

## Abbreviations

ALND: Axillary lymph node dissection
CNB: Core-needle biopsy
CNGOF: Collège National des Gynécologues et Obstétriciens Francais
EORTC: European Organization for Research and Treatment of Cancer
FNA: Fine-needle aspiration
NCCN: National Comprehensive Cancer Network
MDT: Multi-Disciplinary Team conference
MRI: Magnetic resonance imaging
RT: Radiation therapy
SEER: Surveillance, Epidemiology, and End Results Program
SLNB: Sentinel lymph node biopsy
